# Successful Retrieval of a Retained Rectal Foreign Body in the Emergency Department

**DOI:** 10.7759/cureus.2025

**Published:** 2018-01-05

**Authors:** Gabriel O Ologun, Yuan Stevenson, Burt Cagir, Paul Granet, Philip McPhail

**Affiliations:** 1 General Surgery, Robert Packer Hospital/Guthrie Clinic; 2 Colorectal Surgery, Robert Packer Hospital/Guthrie Clinic; 3 Trauma/surgical Critical Care, Robert Packer Hospital/Guthrie Clinic

**Keywords:** rectal foreign body, eroticism, transanal approach, endoscopy, laparotomy

## Abstract

Rectal foreign bodies are a common presenting complaint in the emergency department. Anal eroticism is the major reason for the majority of cases of rectal foreign bodies. A high index of suspicion is required to accurately diagnose a rectal foreign body as patients are often embarrassed about their condition and may not present in a timely fashion to be evaluated or volunteer their history. Extraction techniques include transanal, endoscopic, and laparotomy with repair of complications. Here, we present the case of successful transanal manual removal of a retained dumbbell in the rectum of a middle-aged man.

## Introduction

Rectal foreign bodies are a common presenting complaint in the emergency department with a male-to-female ratio of about 28:1 [1]. Anal eroticism is the major reason for the majority of cases of rectal foreign bodies. Other reasons for anal instrumentation include assault, self-treatment of fecal impaction or prostate massage, and concealment of illicit drugs and weapons [1]. Here, we present the case of a retained dumbbell in the rectum during sexual experimentation in a middle-aged man. Informed consent was obtained for the case report, images, and for publication.

## Case presentation

**Figure 1:**
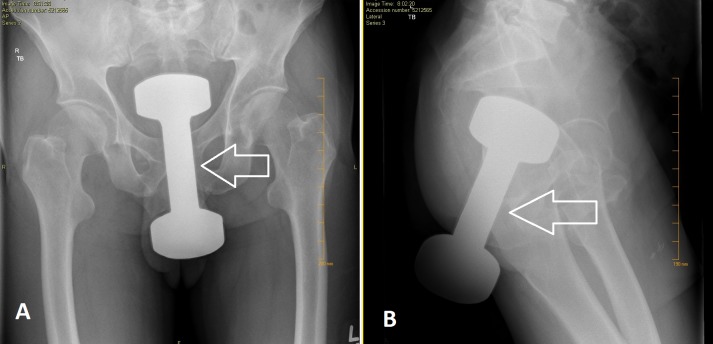
Rectal dumbbell (arrow) on abdominal plain films (A) antero-posterior view and (B) lateral view.

A 50-year-old male presented to the emergency room with complains of lower abdominal pain and discomfort for a four-hour duration while experimenting with new sexual practices. It was caused due to a retained five-pound dumbbell in his rectum. His medical history was unremarkable. An abdominal examination demonstrated mild tenderness in the left lower quadrant. A rectal examination revealed a hard metallic object; no gross blood was found on the examining glove. Blood work showed a normal complete blood count. Biplanar abdominal radiography revealed a radiodense foreign body within the midline of the pelvis consistent with the history of a five-pound weight (Figure 1). The surgical team was consulted.

The patient was placed in the lithotomy position with reverse Trendelenburg angulation. He was under conscious sedation using fentanyl and versed. The anal canal and the examiner's finger were generously lubricated; gentle suprapubic pressure was applied by an assistant to help move the object caudally. The end of the dumbbell was then secured in the lubricated fingers and with slow gentle traction, the object was successfully retrieved. Post extraction, the digital rectal exam revealed a good rectal sphincter tone with minimal blood tinged mucus. He was observed in the emergency room for about six hours. He had no abdominal pain, tolerated oral intake, and voided without difficulty. He was then discharged home.

## Discussion

Patients with a retained rectal foreign body are often ashamed of their diagnosis; they are unable to be completely truthful of the reason for their visit, hence a high index of suspicion is needed for early and accurate diagnosis [2]. Patients typically complain of rectal or abdominal pain, constipation, obstipation, bright red blood per rectum, or incontinence [2-3]. The physical exam should include abdominal exam and a digital rectal exam with a detailed assessment of the anal sphincter. The diagnosis is almost always made with a physical examination (after consulting the history of the patient) and confirmed with a plain radiograph [1]. An erect chest radiograph is usually recommended to rule out rectosigmoid perforation with pneumoperitoneum. A retained rectal foreign body may be classified as high- or low-lying depending on their location relative to the rectosigmoid junction [1]. This is important in the management of the patients as there is evidence to suggest that objects located above the rectum on presentation are more likely to require operative intervention, whereas low-lying foreign bodies that are palpable on a digital rectal exam can be extracted in the emergency department [1-2]. Patients who are peritonitic or hemodynamically unstable should be transported to the operating room for emergent laparotomy without an attempt at bedside extraction of the rectal foreign body [2]. In stable patients, less invasive extraction techniques such as transanal endoscopic extractions can be attempted, while operative extraction is reserved for cases in which the less invasive techniques were not successful [2-3]. Perianal lubrication must be done in all cases [4]. Transanal extraction of rectal foreign bodies has a success rate of 60% to 75%; it is typically performed with local anesthesia with or without conscious sedation [2]. Appropriate sedation including perianal local anesthesia infiltration should be done to facilitate the retraction of the foreign body. The patient should be in the lithotomy position as this is an advantageous position in terms of applying Valsalva maneuver, or abdominal manipulation and abdominal incision, if needed [4]. Some useful tools for grasping objects within reach include ring forceps, obstetric forceps, Kocher clamps, suction devices, and bone cutters. Passing a Foley catheter or a Minnesota tube above the object and then inflating the balloon may break any suction effect and allow traction. Avoid attempts at transanal extraction of sharp objects, rather consider the use of endoscopic techniques or other approaches [2]. Lateral internal sphincterotomy can be performed to aid the removal of a large retained rectal foreign body [4]. Endoscopic extraction technique involves the use of a flexible endoscope to extract objects that are more proximally situated in the rectum or the distal sigmoid colon [3]. It provides great visualization of the mucosa, and a polypectomy snare may be used to help extract the foreign body [2-3]. After successful extraction, the endoscope should be passed again to evaluate the rectal mucosa for any inadvertent injuries [3]. Removal of a sharp item requires endoscopy as well as dragging the sharp point behind, at the time of removal, to prevent injuries [4]. The surgical extraction technique is usually reserved as a last resort after the failure of attempts at transanal removal, or the presence of perforated sepsis or peritonitis [1]. In the absence of perforation, an attempt should be made at milking the object distally into the rectum; if this fails, a laparotomy with colotomy and removal of the foreign body may be indicated [3]. Even when a laparotomy is performed, especially for high foreign objects, colotomy should be avoided and the foreign body should be milked down transabdominally to retrieve it transanally. When indicated, colotomy should be placed at the rectosigmoid junction or in the upper rectum if needed [4]. In the presence of gross contamination, a Hartmann procedure may be advisable; however, a primary repair of the short segment resection may be performed in an otherwise non-contaminated field with a viable bowel wall [1-2]. Inspect the rest of the bowel for any additional injuries prior to closing the abdomen [3]. Post the extraction, patients are observed for hours to days depending on several factors including the clinical status of the patient, comorbidities, delay in presentation, and whether or not trauma to the rectum or surrounding tissue was present [1]. The goal is for early detection of complications such as perforation, which can be evaluated with computed tomography (CT) scans of the abdomen/pelvis with rectal contrast [2]. Sphincter function should be assessed and documented post extraction, as traumatic disruption of the anal sphincter can result in mild to severe fecal incontinence; however, many of these improve with observation alone. A follow-up period of at least three months may be recommended before considering attempts at sphincter repair [2-3].

## Conclusions

Rectal foreign bodies are a common presenting complaint in the emergency department. Anal eroticism is the major reason for the majority of cases of rectal foreign bodies. A high index of suspicion is required for timely and accurate diagnosis. Extraction techniques include transanal, endoscopic, and laparotomy. Operative extraction is reserved for cases in which the less invasive techniques are not successful; however, it may be the initial approach in patients with a delayed presentation and who are peritonitic or hemodynamically unstable.

## References

[1] Ayantunde AA (2013). Approach to the diagnosis and management of retained rectal foreign bodies: clinical update. Tech Coloproctol.

[2] Cologne KG, Ault GT (2012). Rectal foreign bodies: what is the current standard?. Clin Colon Rectal Surg.

[3] Goldberg JE, Steele SR (2010). Rectal foreign bodies. Surg Clinc North Am.

[4] Lake JP, Essoni R, Petrone P (2004). Management of retained colorectal foreign bodies: predictors of operative intervention. Dis Colon Rectum.

